# Emotional and rational disease acceptance in patients with depression and alcohol addiction

**DOI:** 10.1186/1477-7525-6-4

**Published:** 2008-01-21

**Authors:** Arndt Büssing, Peter F Matthiessen, Götz Mundle

**Affiliations:** 1 Chair of Medical Theory and Complementary Medicine, University Witten/Herdecke, Gerhard-Kienle-Weg 4, 58313 Herdecke, Germany; 2 Oberberg Klinik Schwarzwald, Oberberg 1, 78132 Hornberg, Germany

## Abstract

**Background:**

The concept of a rational respectively emotional acceptance of disease is highly valued in the treatment of patients with depression or addiction. Due to the importance of this concept for the long-term course of disease, there is a strong interest to develop a tool to identify the levels and factors of acceptance. We thus intended to test an instrument designed to assess the level of positive psychological wellbeing and coping, particularly emotional disease acceptance and life satisfaction

**Methods:**

In an anonymous cross-sectional survey enrolling 115 patients (51% female, 49% male; mean age 47.6 ± 10.0 years) with depression and/or alcohol addiction, the ERDA questionnaire was tested.

**Results:**

Factor analysis of the 29-item construct (Cronbach's alpha = 0.933) revealed a 4-factor solution, which explained 59.4% of variance: (1) Positive Life Construction, Contentedness and Well-Being; (2) Conscious Dealing with Illness; (3) Rejection of an Irrational Dealing with Disease; (4) Disease Acceptance. Two factors could be ascribed to a rational, and two to an emotional acceptance. All factors correlated negatively with Depression and Escape, while several aspects of Life Satisfaction" (i.e. myself, overall life, where I live, and future prospects) correlated positively. The highest factor scores were found for the rational acceptance styles (i.e. Conscious Dealing with Illness; Disease Acceptance). Emotional acceptance styles were not valued in a state of depression. Escape from illness was the strongest predictor for several acceptance aspects, while life satisfaction was the most relevant predictor for "Positive Life Construction, Contentedness and Well-Being".

**Conclusion:**

The ERDA questionnaire was found to be a reliable and valid assessment of disease acceptance strategies in patients with depressive disorders and drug abuses. The results indicate the preferential use of rational acceptance styles even in depression. Disease acceptance should not be regarded as a coping style with an attitude of fatalistic resignation, but as a complex and active process of dealing with a chronic disease. One may assume that an emotional acceptance of disease will result in a therapeutic coping process associated with higher level of life satisfaction and overall quality of life.

## Background

Among the numerous ways to cope with disease, two general strategies can be distinguished: 1. problem-solving (i.e. do something active to avoid stressful circumstances) and 2. emotion-focused coping strategies (i.e. try to regulate the emotional consequences of stressful or potentially stressful events). Folkman and Lazarus [1] found that both types are used to face stressful situations. In contrast, Carver et al. [2] found 15 factors that reflect active versus avoidant coping strategies, among them "Resignation/Acceptance" (accepting the fact that the stressful event has occurred and is real) and "Focus on and Venting of Emotions" (increased awareness of one's emotional distress, and concomitant tendency to ventilate or discharge those feelings).

An active coping means to change the nature of the stressor itself or how one thinks about it. In contrast, avoidant strategies are intended to prevent a direct confrontation with the stressful events, and may often result in inappropriate activities such as alcohol abuse or depressive states. These avoidance strategies were identified as psychological risk factors or marker for adverse responses to stressful life events [3]. Data from depressed patients showed that a better clinical course of depression was associated with patients who had high levels of social support, had more active and less avoidant coping styles, and who were physically active [4]. Lung transplant candidates most likely use active, acceptance, and support-seeking strategies to cope with health problems, while self-blame or avoidance were rarely used [5]; however, the avoidant coping was the most strongly and consistently related to quality of life.

Evers et al. [6] proposed three generic illness cognitions that reflect different ways of re-evaluating the inherently aversive character of chronic disease: 1. helplessness as a way of emphasizing the aversive meaning of disease, 2. acceptance as a way to diminish the aversive meaning, and 3. perceived benefits as a way of adding a positive meaning to the disease. However, according to Carver's conceptualizations [2], disease acceptance has often a connotation of resignation and fatalism. In fact, in patients with rheumatoid arthritis, illness acceptance beliefs were identified as significant predictors of both anxiety and depression [7].

Among several psychosocial factors associated with depression and/or stress resilience [8], one may find positive emotions and optimism, cognitive flexibility, cognitive explanatory style and reappraisal, acceptance, and religion/spirituality. Intensive research during the last decades has brought a shift from a somatic determined acceptance of disease and disorders to a more psychological perspective. In fact, most chronic diseases are influenced by somatic, psychological, social, and spiritual factors, and thus an exclusive focus on the somatic or solely the psychological aspects is a short-cut rather than a comprehensive approach which acknowledges the multi-factorial aetiology of chronic disease and the complex process-oriented therapeutic approaches. Education and training help patients to develop living patterns that incorporate self-management. According to this informative strategy, the patients learn about causes and sources, and what to focus on and what to ignore. This is a cognitive (rational) based strategy to deal with chronic illness. Although education and self-management are significant aspects of treatment, however, several patients with depression or addiction experience recurrent failure despite of this knowledge. To achieve a long-lasting and thus effective treatment, the emotional acceptance of disease with handling of feelings of anger, guilt or escape and integration of the disease as a permanent 'note' into the self-concept, is of out-standing importance. In fact, in out-patients with schizophrenia, Cooke et al. [9] demonstrated that "awareness of symptoms and problems" correlated with greater distress, while "preference for positive reinterpretation and growth" was associated with lower distress and symptom awareness (re-labelling), and "social support-seeking" with greater awareness of illness, but not distress [9].

In the Oberberg Concept, which was developed by Professor Matthias Gottschaldt in the early 1980s [10-12], the concept of a rational and emotional acceptance of the disease is highly valued in the treatment of patients with depression or addiction. The 'Oberberg Concept' postulates, that the rational and especially the emotional acceptance are important coping strategies to prevent relapse. Unaware emotional non-acceptance of the disease by the patient, such as denial, guilt, fighting against or escape of the disease, are believed to be significant risk factors for relapse even if the patient is able to accept his disease rationally. At the beginning of the therapeutic process, the patient is often unaware of his dysfunctional emotional coping strategies. At this initial stage, the patient feels unconsciously angry and defensive, as well as guilty or shameful for the development of the disease. According to the 'Oberberg Concept'[10-12], one has to focus on recognizing these individual dysfunctional emotional coping strategies. Through daily individual and group therapy sessions, the patient mindfully learns to recognize his functional and dysfunctional emotional coping strategies and their origins. These origins are most often a combination of current conflicts and imprints of childhood memories, which are mainly unconscious. If the disease is emotionally accepted, the patient is able to see his disease as a medical condition and not as a personal failure, and thus will be able to accept the necessary current and future treatment as well as learning how to adequately deal with difficulties caused by the disease. If the patient still fights emotionally against the disease, early warning symptoms of a relapse will not be recognized and necessary treatment will not be chosen even if the patient knows rationally all treatment options.

Due to the importance of the rational and emotional acceptance upon the treatment and long-term course of the disease, there is a strong interest to develop a tool to identify the levels and factors of acceptance. The intention of this work was thus to develop and test a new instrument designed to assess the level of positive psychological wellbeing and coping, particularly emotional disease acceptance and life satisfaction, in patients with depressive s and addictive behaviour pattern.

## Methods

### Procedure and subjects

All individuals of this cross-sectional anonymous survey were informed of the purpose of the study, were assured of confidentiality, and gave informed consent to participate. The patients were recruited consecutively in three German clinics, i.e. Oberberg Clinics Schwarzwald, Weserbergland, and Berlin/Brandenburg. The private specialist emergency clinics within the Oberberg group offer comprehensive medical and psychotherapeutic treatment for individuals suffering from emotional, psychosomatic and psychiatric problems, such as addictive behaviour patterns, depression, and burn-out.

All subjects completed the anonymized questionnaire, which did not ask for name or for initials, by themselves. Moreover, all anonymous questionnaires were stored 470 km away from the clinics at the University Witten/Herdecke, and were transferred into an electronic data pool. A later allocation of the data to concrete patients is thus impossible.

The sample of this cross-sectional survey contained 115 patients (51% female, 49% male) with a mean age of 47.6 ± 10.0 years. 49% had a depression (or associated diseases, i.e. burn out, anxiety disorders), 24% alcohol addiction (just 3 patients with others addictions), 12% depression and addiction, and 16% diseases which were within the unique therapeutic context of the respective clinics, i.e. addictive behaviour patterns, depression, and burn-out, but not specified by the patients.

Although depression and alcohol abuse are separate but often co-morbid disorders with different aetiologies, trajectories and consequences, the therapeutic concepts of the Clinics nevertheless focus on emotional disease acceptance as an integral aspect of an active therapeutic process. One may suggest that several of them have used avoidance strategies in their past.

Most of the patients were married (45%), 14% were living with a partner not married with, 17% were divorced, 23% living alone, and 1% widowed. Sixty-four% had a high school education (Gymnasium), 19% a secondary education (junior high; Realschule), 4% a secondary education (Hauptschule), and 13% other. Most of them had a Christian affiliation (68%), 31% none, and 1% other. Fifty-five% were employees, 26% self-employed, 6% house wives/men, 4% unemployed, 9% in early retirement, and 2% incapacitated. With respect to these variables, no significant differences were found between the disease groups (data not shown).

As shown in table 1, the disease groups did significantly differ in terms of depression index, Escape, life satisfaction and attendance of a support group, while for age and mean duration of disease just a remarkable trend was observed. In fact, patients with depression and related diseases of course had a higher depression index, Escape and lower life-satisfaction, while patients with addictions of course attended support groups more frequently, had a longer duration of disease, were older, and, however, had a higher life-satisfaction.

**Table 1:** Demographic and psychological data of 115 patients

	**All patients**	**depression**	**alcohol addiction**	**addiction and depression**	**unspecified diseases**	**p-value**^1^
**Number**	115	55	25	14	17	

**Gender**						n.s
female (%)	51	60	44	36	47	
male (%)	49	40	56	64	53	

**Mean age **(years)	47.6 ± 10.0	45.1 ± 11.0	51.4 ± 8.8	48.7 ± 10.0	48.8 ± 6.8	0.054

**Mean duration of disease **(months)	46.1 ± 64.1	33.8 ± 52.0	54.1 ± 69.9	83.9 ± 86.5	/	0.073

**Beck Depression Index**	14.5 ± 10.5	18.4 ± 10.8	8.6 ± 8.0	12.9 ± 7.1	12.2 ± 7.1	**0.001**

**Escape Score **^2^	47.0 ± 27.4	58.4 ± 19.6	37.0 ± 23.3	45.8 ± 28.4	39.8 ± 30.1	0.027

**Life-Satisfaction **^3^	63.5 ± 19.9	58.4 ± 19.6	71.5 ± 17.8	62.9 ± 25.1	68.1 ± 15.5	0.026

**Support group attendance**						**0.000**
Never (%)	69	92	38	25	71	
rarely/irregularly (%)	19	6	38	33	24	
regularly (%)	12	2	26	42	6	

^1 ^cross-tabulation (Chi2) and ANOVA, respectively

^2 ^Sum Score – Escape from illness [13].

^3 ^Sum Score – Brief Multidimensional Life Satisfaction Scale modified according to Huebner [15] with two additional items.

### Measures

The items of the ERDA (acronym of "Emotional/Rational Disease Acceptance") questionnaire were developed with the input of patients and experts, particularly statements of psychiatrists, psychologists, and other therapists from the Oberberg clinics. On the basis of the expertise of the three heads of the Oberberg clinics, 48 items were chosen among a sample of several others suggested to address the underlying concept of an emotional respectively rational disease acceptance.

All items were scored on a 5-point scale from disagreement to agreement (0 – does not apply at all; 1 – does not truly apply; 2 – don't know; 3 – applies quite a bit; 4 – applies very much). Some items were recoded because of an intended negative direction (indicated in table 2 with "-"). The final scores were referred to a 100% level (4 "applied very much" = 100%).

**Table 2:** Mean values and reliability parameters

	**mean**	**SD**	**Difficulty index ** (0.60)	**loading**	**corrected item-total correlation**	**alpha if item deleted** (Cronbach's alpha = 0.933)
**Positive Life Construction, Contentedness and Well-Being *(emotional)***						
K5 it works to manage life by myself despite of symptoms	2.64	1.27	0.66	.732	.662	.929
K3 can do all which is important to me despite of symptoms	2.12	1.48	0.53	.832	.664	.929
K6 even when negative emotions will appear, I don't let them control me	2.13	1.23	0.53	.655	.574	.930
K2 come to grips with daily life despite of symptoms	2.37	1.27	0.59	.846	.650	.930
K4 can't get on with the impacts of disease (-)	2.21	1.35	0.55	.661	.685	.929
K24 feel well (inside)	1,84	1.30	0.46	.620	.703	.929
K11 understand the causes of disease, but I don't get on with it (-)	2.19	1.36	0.55	.570	.698	.929
K26 comfortable with myself and my situation	1,48	1.28	0.37	.764	.599	.930
K7 life is centred by disease (-)	2.52	1.32	0.63	.736	.596	.930
K28 it saddens that disease has destroyed so much in my life	1.66	1.37	0.42	.506	.448	.932
K37 can live with the fact that disease may reappear in stressful situations	2.16	1.29	0.54	.592	.614	.930

**Conscious Dealing with Illness *(rational)***						
K35 aware of the consequences of my disease for myself and family, and thus I have the unconditional will to work on myself	3.25	1.00	**0.81**	.839	.424	.932
K15 due to the accepting handling of my disease, I have got a better understanding to deal with the troubles in life	2.71	1.18	0.68	.792	.405	.932
K16 do know that I have to live with my disease and have to care each and every to prevent the reappearance of its impacts	3.07	1.05	0.77	.778	.329	.933
K33 even when relapses may occur, I have the unconditional will to work on my recovery further on	3.37	0.98	**0.84**	.744	.421	.932
K34 do know that I have to live with disease, but I don't want it anyhow (-)	2.49	1.46	0.62	.640	.610	.930
K22 the role the disease plays in the handling of my emotions is clear to me	2.82	1.07	0.71	.562	.371	.933

**Rejection of an Irrational Dealing with Disease *(emotional)***						
K30 feel guilty to be ill (-)	2.49	1.45	0.62	.582	.650	.929
K41 feels highly incorrect to regard a disease as the cause of my afflictions rather than myself as a person (-)	2.39	1.30	0.60	.685	.376	.933
K39 sometimes I would like to wake up and ascertain that I never had been ill (-)	1.56	1.43	0.39	.640	.457	.932
K43 idea that my symptoms and troubles arise from a disease seems as an incapacitation (-)	2.80	1.20	0.70	.665	.484	.932
K29 when ill, feeling of failure (-)	1.80	1.44	0.45	.515	.608	.930
K45 it annoys me that disease will come along with me my whole life (-)	1.34	1.37	0.34	.560	.380	.933

**Disease Acceptance *(rational)***						
K9 can understand the causes of my disease	2.87	1.14	0.72	.794	.395	.932
K8 do know that I am ill and can accept it	2.80	1.26	0.70	.791	.533	.931
K12 understand the causes of my disease, but don't find an emotional access to them (-)	2.47	1.25	0.62	.733	.568	.931
K13 disease is apart of me which I can't accept (-)	2.51	1.45	0.63	.451	.578	.930
K25 fail to accept my disease (-)	2,78	1.25	0.70	.416	.691	.929
K38 accept disease as a part of me	2.47	1.25	0.62	.502	.710	.929

SD – standard deviation; DI – difficulty index; (-) – items with a negative statement were recoded

Extraction of the main components eigenvalue > 1); Varimax Rotation with Kaiser

Normalization (rotation converged in 7 Iterations).

For external correlations, we used the Beck-Depression-Index (BDI), the Escape scale (Büssing et al., 2006) which measures an attitude of depressive escape from illness ("fear what illness will bring", "would like to run away from illness", "when I wake up, I don't know how to face the day"); moreover, the AKU questionnaire which measures six different adaptive coping styles [13,14], the Brief Multidimensional Life Satisfaction Scale according to Huebner [15] with two additional items, and Meaning of Illness according to Lipowski [16,17].

### Statistical analysis

Reliability and factor analyses of the inventory were performed according to the standard procedures as described previously [17]. To combine several items with similar content, we relied on the technique of factor analysis which examines the correlations among a set of variables, and to achieve a set of more general "factors". Factor analyses (extraction of main components with eigenvalues > 1) were repeated rotating different numbers of items (Varimax rotation with Kaiser Normalization) in order to arrive at the solution which demonstrates both the most simple and the most coherent structure.

All reliability and factor analyses, analyses of variance (ANOVA), correlation analyses, and tests of between-subjects effects were performed with SPSS for Windows 12.0. We judged p < 0.05 significant, and 0.05 < p < 0.10 as a trend.

## Results

### Reliability

In order to eliminate items from the 48-item pool that were not contributing to the questionnaire reliability, items which were too complicated in the phrasing or with a poor reliability (<0.2) had to be removed, among them 3 items which would make up a scale termed "Appreciation and Gratitude". As shown in table 2, the resulting 29-item construct had a good quality (Cronbach's alpha = 0.933). The item difficulty (2.38 [mean value]/4) was 0.60. With the exception of item K35 (0.81) and item K33 (0.84) which tended to have a ceiling effect, all values were in the acceptable range from 0.2 to 0.8.

### Factor analysis

Factor analysis of the questionnaire revealed a Kaiser-Mayer-Olkin value of 0.840, which as a measure for the degree of common variance, indicates that the item-pool is suitable for a factorial validation.

Primary factor analysis pointed to a 6-factor solution (all with initial eigenvalues > 1), which would explain 67.4% of variance: a 6-item sub-scale "Arrangement with Symptoms and Positive Life Construction"; a 6-item sub-scale "Conscious Dealing with Illness"; a 7-item sub-scale "Dealing with Irrational Disease Rejection"; a 5-item sub-scale "Contentedness and Well-Being (despite of Disease)"; a 3-item sub-scale "Rational Disease Acceptance"; and a 4-item sub-scale "Emotional Disease Rejection".

Due to the fact that the tentative factors 5 and 6 consist of just 3 or 4 items, we favoured a 4-factor solution, which explains 59.4% of variance (Table 2): The strongest factor with an eigenvalue of 10.4, termed "Positive Life Construction, Contentedness and Well-Being" is made up by 11 items of the former factors 1 and 4, and had an alpha of 0.921. The 6-item sub-scale "Conscious Dealing with Illness" with an eigenvalue of 3.3 had an alpha of 0.778. The sub-scale "Dealing with Irrational Disease Rejection" with 6 negative statements (which were recoded) and an eigenvalue of 2.0, had an alpha of 0.766, while the 6-item sub-scale "Disease Rejection" with an eigenvalue of 1.5 was made up by the former factors 5 and 6, and had an alpha of 0.843. Thus, the internal consistency of the item pool was sufficiently high.

Analysis of the secondary loadings (only values > 0.45 were take into account) revealed that item K29 from factor 3 would also load on factor 1 (0.539), and item K34 from factor 2 also on factor 3 (0.503).

### Correlation analyses

We found several relevant correlations between the factors of the instrument (Table 3). While the more emotion-associated factors such as "Positive Life Construction, Contentedness and Well-Being" and "Rejection of an Irrational Dealing with Disease" correlated well together (r = 0.539), particularly the factor "Conscious Dealing with Illness" which is a cognitive style correlated just moderately with the emotional factors (r < 0.44). However, the factor "Disease Acceptance" correlated with all other factors, and best with the rational style "Conscious Dealing with Illness" (r = 0.598).

**Table 3:** Correlations between the disease acceptance factors

	**Positive Life Construction, Contentedness and Well-Being**	**Conscious Dealing with Illness**	**Rejection of an Irrational Dealing with Disease**	**Disease Acceptance**
Positive Life Construction, Contentedness and Well-Being	1	.439	**.539**	**.503**
Conscious Dealing with Illness		1	.351	**.598**
Rejection of an Irrational Dealing with Disease			1	**.512**
Disease Acceptance				1

All correlations are significant at the 0.01 level (2-tailed Pearson Correlation).

All 4 factors correlated negatively with the Beck-Depression-Inventory and the Escape scale, particularly "Positive Life Construction, Contentedness and Well-Being" (Table 4). In contrast, several aspects of life satisfaction" (i.e. myself, overall life, where I live, and future prospects) correlated positively with the disease acceptance factors, again "Positive Life Construction, Contentedness and Well-Being" revealed the strongest associations. The "future prospects" did correlate strongly with "Positive Life Construction, Contentedness and Well-Being" and "Conscious Dealing with Illness" (r > 0.5). Thus, the observed correlations are plausible and support external validity of the construct.

**Table 4:** Correlations of disease acceptance with external factors

	**Positive Life Construction, Contentedness and Well-Being**	**Conscious Dealing with Illness**	**Rejection of an Irrational Dealing with Disease**	**Disease Acceptance**
**Depression**				
BDI	-.656 **	-.430 **	-.483 **	-.413 **
Escape	-.685 **	-.510 **	-.611 **	-.574 **

**Life Satisfaction **^1^				
*Sum-Score*	.727 **	.459 **	.401 **	.405 **
family life	.494 **	.300 **	.214 *	.172
friendships	.427 **	.252 **	.237 *	.171
work	.350 **	.266 **	.231 *	.228 *
myself	.713 **	.421 **	.517 **	.515 **
where I live	.547 **	.286 **	.282 **	.247 **
overall life/life in general	.659 **	.409 **	.339 **	.387 **
financial situation	.495 **	.241 **	.253 **	.263 **
future prospects	.626 **	.561 **	.321 **	.457 **

**Meaning of Illness **^2^				
challenge	.332**	.371 **	.261 **	.375 **
threat/enemy	-.273 **	-.101	-.324 **	-.219 *
adverse interruption	-.201 *	-.236 *	-.317 **	-.233 *
punishment	-.366 **	-.235 *	-.424 **	-.335 **
weakness/failure	-.453 **	-.214 *	-.525 **	-.423 **
value	.113	.380 **	.031	.207 *
relieving break	-.116	.089	-.193 *	.004
cry for help	-.193 *	.215 *	-.214 *	.209 *

**Adaptive Coping Styles **^3^				
Trust in God's help	.174	.403 **	.115	.156
Conscious and Healthy Living	.375 **	.696 **	.118	.390 **
Reappraisal: Illness as Chance	-.008	.324 **	-.032	.248 **
Perspectives & Positive Attitudes	.461 **	.641 **	.210 *	.521 **
Trust in Medical Help	.026	.360 **	.111	.340 **
Search for Alternative Help	.266 **	.471 **	.040	.391 **

Pearson correlations are significant at the ** 0.01 respectively the * 0.05 level (2-tailed).

^1 ^Brief Multidimensional Life Satisfaction Scale modified according to [15] with two additional items.

^2 ^Meaning of Illness according to Lipowski [16,17] ^3 ^Adaptive Coping Styles as measured with the AKU questionnaire [13,14]

The correlation analyses between "Meaning of Illness" and the ERDA factors revealed differential pattern. The rational factor "Conscious Dealing with Illness" correlated well with positive disease interpretations such as "challenge" and "value" (r > 0.35), while particularly "value" did not correlate significantly with the emotional styles. On the other hand, "weakness/failure" correlated negatively with the emotional factors "Positive Life Construction, Contentedness and Well-Being" and "Rejection of an Irrational Dealing with Disease". In contrast, disease interpretation as a "relieving break" did not correlate with the disease acceptance factors (just a minor correlation with "Rejection of an Irrational Dealing with Disease"); also "Cry for help" showed just some minor correlations with the acceptance styles. This means, the differential pattern of disease interpretation and acceptance are plausible from a theoretical point of view, too.

With respect to the adaptive coping styles, we found a strong correlation between "Conscious Dealing with Illness" and "Conscious and Healthy Living" (r = 0.696) and with "Perspectives and Positive Attitudes" (r = 0.641). With the exception of "Conscious Dealing with Illness" (r = 0.388), none of the disease acceptance factors did correlate with "Trust in God's Help".

In accordance with previous findings that the factor "Reappraisal: Illness as Chance" can be interpreted as an unique spiritual attitude [14,17], this factor correlated with "Conscious Dealing with Illness" too (r = 0.324). This unique scale ("Conscious Dealing with Illness") correlated also with "Search for Alternative Help" (0.471), "Trust in Medical Help" (r = 0.360), with disease interpretations "challenge" (r = 0.371) and "value" (r = 0.380), and with life satisfaction aspect "future prospects" (r = 0.561).

### Factor scores

Over all, the highest assent was found for the factors "Conscious Dealing with Illness" and "Disease Acceptance", both more rational styles of disease acceptance, while the lowest assent score were found for "Rejection of an Irrational Dealing with Disease" (Table 5).

**Table 5:** Mean score values

		**Positive Life Construction, Contentedness and Well-Being**	**Conscious Dealing with Illness**	**Rejection of an Irrational Dealing with Disease**	**Disease Acceptance**
**All patients**	Mean	51.03	76.08	48.98	66.19
(n = 115)	SD	23.69	18.17	22.64	23.37

**Disease group**					

Depression	Mean	44.60	72.06	45.94	61.58
(n = 67)	SD	23.21	19.12	22.38	22.15
Addictions	Mean	58.65	85.31	**56.54**	74.20
(n = 27)	SD	22.98	13.16	19.60	20.93
Depression + addictions	Mean	50.65	80.54	**41.37**	72.92
(n = 14)	SD	22.06	19.20	21.59	24.34
Not specified	Mean	**59.90**	71.25	53.01	63.29
(n = 15)	SD	22.95	15.95	26.23	27.07
F-value		3.345	**4.280**	2.119	2.344
p-value		0.022	**0.007**	n.s.	0.077

**Depression**					

BDI < 12	Mean	**61.90**	82.48	**57.28**	73.19
(n = 53)	SD	20.32	15.04	21.67	23.49
BDI > 12	Mean	**38.96**	71.72	**40.95**	60.85
(n = 51)	SD	21.53	19.65	20.25	21.34

F-value		**31.266**	**9.876**	**15.744**	**7.847**
p-value		**0.000**	**0.002**	**0.000**	**0.006**

**Escape**					

< 50%	Mean	**60.67**	81.91	**57.83**	74.75
(n = 70)	SD	20.72	16.33	19.90	19.11
>50%	Mean	**36.03**	67.00	**35.20**	52.87
(n = 45)	SD	20.06	17.28	19.71	23.32

F-value		**39.713**	**21.814**	**35.681**	**30.161**

p-value		**0.000**	**0.000**	**0.000**	**0.000**

**Attendance of support group**					

Never	Mean	47.24	71.24	45.91	60.96
(n = 87)	SD	22.65	18.26	22.58	23.20
Seldom/infrequently	Mean	51.90	80.54	45.71	65.21
(n = 20)	SD	27.91	17.92	20.71	23.35
Regularly	Mean	**64.27**	**87.56**	**63.78**	**86.79**
(n = 13)	SD	22.76	11.98	18.74	12.20

F-value		2.904	**5.983**	3.841	**7.457**
p-value		0.059	**0.003**	0.025	**0.001**

BDI – Beck Depression Index

Deviations > 15% from the mean were highlighted

There were several highly significant differences with respect to disease group and attendance of a support group (Table 5), i.e. significantly higher scores of the more cognitive styles were found in patients attending a support group regularly. However, there were no significant differences with respect to gender, educational level, and duration of disease (data not shown).

Significant differences were found also for "Positive Life Construction, Contentedness and Well-Being"; the scores were highest in elderly (F = 3.601; p = 0.016), married patients (F = 2.481; p = 0.048), and in those with a Christian affiliation rather than none (F = 5.306; p = 0.006). Moreover, higher scores of "Conscious Dealing with Illness" were found in those with a religious affiliation (F = 4.496; p = 0.013), and in self-employed and house-wives/men rather than employees, unemployed or incapacitated (F = 2.379; p = 0.044).

The most relevant variables which could explain the major differences in the factor scores were the Beck Depression Index and the Escape score (Table 5). Patients without depression (BDI = 12) and low Escape (<50%) had the highest disease acceptance scores (p < 0.01).

### Predictors of disease acceptance

To determine predictors of the disease acceptance aspects, we performed stepwise regression analyses. The following variables emerged: depression (BDI), Escape, life-satisfaction, adoptive coping styles (AKU, i.e. Trust in God's help, Conscious and Healthy Living, Reappraisal: Illness as Chance, Perspectives & Positive Attitudes, Trust in Medical Help, Search for Alternative Help), family status, disease group, and attendance of a support group.

As shown in table 6 for the factor "Positive Life Construction, Contentedness and Well-Being", the regression model 1 was able to explain 50% of variance (R^2^), while an investigation of the standardized beta coefficients show that the parameter life satisfaction had the highest influence, followed by parameters Escape, educational level, depression, and family status.

**Table 6:** Predictors of disease acceptance aspects (regression model)

**Factor**	Predictors*	R^2^*	**B**	**Std. Err.**	**Beta**	**T**	**Sign. T**
Positive Life Construction, Contentedness and Well-Being	(constant)	.497	68.508	12.754		5.372	.000
	Life Satisfaction		.426	.106	.354	4.033	.000
	Escape		-.333	.072	-.370	-4.618	.000
	Educational level		-5.544	2.422	-.148	-2.289	.025
	Beck-Depression-Index		-.524	.213	-.222	-2.462	.016
	Family Status		-2.789	1.270	-.145	-2.196	.031

Conscious Dealing with Illness	(constant)	.388	56.597	7.621		7.426	000
	Escape		-.260	.050	-.426	-5.148	.000
	Conscious and Healthy Living		.449	.084	.443	5.361	.000

Rejection of an Irrational Dealing with Disease	(constant)	.421	85.967	11.343		7.579	.000
	Escape		-.496	.080	-.608	-6.203	.000
	Beck-Depression-Index		-.564	.198	-.264	-2.849	.006
	Search for Alternative Help		-.412	.127	-.308	-3.241	.002
	Trust in Medical Help		.305	.124	.216	2.467	.016

Disease Acceptance	(constant)	.411	55.532	11.103		5.002	.000
	Escape		-.394	.077	-.464	-5.137	.000
	Perspectives & Positive Attitudes		.376	.121	.276	3.105	.003
	Attendance of Support Group		6.251	2.542	.196	2.459	.016

B, factor B; Beta, beta coefficient; Std Err, standard error of B; T, t-test; sign. T significance (T)

* only the strongest prediction model was presented

For the factor "Conscious Dealing with Illness", the standardized beta coefficients indicate that Escape was the strongest predictor, followed by Conscious and Healthy Living (Table 6).

Escape had the strongest influence also on the factor "Rejection of an Irrational Dealing with Disease", followed by depression, Search for Alternative Help, and Trust in Medical Help (Table 6).

With respect to "Disease Acceptance", an investigation of the standardized beta coefficients show that again Escape had the strongest influence, followed by the parameters Perspectives and Positive Attitudes, and Attendance of Support Group (Table 6).

Given the importance of this Escape factor and to clarify it's inter-correlations, we confirmed that Escape correlated strongly with depression (r = 0.562) and negatively with life satisfaction (r = -0.566); among the adaptive coping styles, it correlated negatively with "Perspectives and Positive Attitudes" (r = -0.480) and "Conscious and Healthy Living" (r = -0.378); and with disease interpretation "weakness/failure" (r = 0.468), "punishment" (r = 0.412), "threat/enemy" (r = 0.326), and negatively with "challenge" (r = -0.321).

## Discussion

Among the items intended to address emotional and more rational styles of disease acceptance, we defined four (respectively six) different factors with eigenvalues > 1: Two factors could be assigned to emotional styles of disease acceptance, i.e. "Positive Life Construction, Contentedness and Well-Being" and "Rejection of an Irrational Dealing with Disease", which both would value a positive attitude to manage life despite of disease, while the factor "Conscious Dealing with Illness" clearly addresses a rational aspect of disease acceptance. However, the factor "Disease Acceptance" could be sub-divided in two factors, each with < 5 items, i.e. a more emotional and a rational factor respectively. Due to this fact, the factor correlated strongly with the other three factors.

We found strong correlations between the emotional styles which are plausible in the light of the underlying construct. It is obvious that the concepts of emotional and rational disease acceptance are different from a theoretical point of view, but nevertheless, there are interconnected.

Although all 4 factors correlated negatively with depression and escape from illness, we found unique disease acceptance pattern. Particularly the highly valued factor "Conscious Dealing with Illness" correlated strongly with an internal adaptive coping (i.e. "Perspectives and Positive Attitudes" and "Conscious and Healthy Living"), with the life satisfaction aspect "future prospects"; which means, that a rational acceptance is an conscious and active process of conduct of life. In contrast, the moderately valued factor "Positive Life Construction, Contentedness and Well-Being" correlated strongly with several life satisfaction aspects, and just moderately with "Perspectives and Positive Attitudes". Although "Rejection of an Irrational Dealing with Disease" can be regarded as a more emotional acceptance style, it differs from the pattern of the other emotional factor, and correlated negatively with disease interpretation "weakness", and life satisfaction aspect "myself".

It was striking that patients in a state of depression respectively escape nevertheless had higher scores in the factors reflecting rational disease acceptance, but low scores in the factors representing an emotional acceptance. The depression state factor Escape was found to be the most important predictor for several disease acceptance aspects, while life satisfaction was the strongest predictor for "Positive Life Construction, Contentedness and Well-Being". Therefore, it would be interesting to follow the courses of the patients during the therapeutic intervention. It remains to be clarified whether an increase of emotional acceptance scores within time may indicate lower frequency of relapses and could thus be used as a marker for an effective treatment.

In agreement with findings of others [18] that physical factors such as gender, age, disease duration and severity of disease had no effect on acceptance of illness, we also did not find significant effects of gender, educational level, and duration of disease, but of higher age, family status (married patients) and religious affiliation. Multivariate analyses revealed a complex pattern of influencing variables, particularly a depressive escape from illness and life satisfaction.

In patients with chronic Psoriasis vulgaris, higher levels of optimism, lower conviction of others' influence on one's health and the less frequently employed coping strategy "concentration on emotions" were correlated with higher acceptance of disease [18]. Although we investigated a completely different set of patients than Zalewska and co-workers [18], we do suggest that the concept of a rational/emotional disease acceptance goes far beyond fatalistic resignation. Based on the results from correlation analysis, the rational factor "Conscious Dealing with Illness" which revealed the highest scores at all (particularly in patients with addictions and patients attending a support group), reflects a strong will of the patients to respond to the challenges of life and disease, to behave more consciously, with an expectancy of positive future prospects, but also reliance on external sources of help. It seems that this factor is of outstanding importance too, and could be the headstone of an effective treatment.

Literature data value the factor optimism as crucial for physical and psychological well-being and resistance towards stressful life events [19]. Although not identical, life satisfaction was the strongest predictor for the emotional factor "Positive Life Construction, Contentedness and Well-Being", which correlated negative with depression and escape, and positive with "Perspectives and Positive Attitudes". This could be interpreted as an active management of life and appearing problems with a sense of fighting spirit. In deed, optimism and self-mastery were found to be empirically distinct, although substantially correlated constructs [20]. In postpartum depression, self-esteem and not optimism appeared to be a reliable contributing factor to the differential susceptibility to depression [21]. – And from a conceptual point of view, self-esteem seems to be much more related to "Positive Life Construction, Contentedness and Well-Being" than optimism.

## Conclusion

Our results confirmed that the instrument is a reliable and valid assessment of disease acceptance strategies in patients with depressive disorders and alcohol abuses. Moreover, the results indicate the preferential use of rational acceptance styles even in depression. Disease acceptance should not be regarded as a coping style with an attitude of fatalistic resignation, but as a complex and active process of dealing with a chronic disease. An important fact which underlines the differential use of these disease acceptance styles is that emotional acceptance was not valued in depression, but in the absence of depression and escape from illness. Although in patients with rheumatoid arthritis, illness acceptance beliefs were identified as significant predictors of both anxiety and depression [7], in our study all disease acceptance aspects correlated strongly with life satisfaction, and negatively with depression and escape. One may assume that an emotional acceptance of disease rather than just a rational acceptance will result in a therapeutic process of disease coping associated with higher level of life satisfaction and overall quality of life. But this remains to be proven in a further study.

Next, the instrument has to undergo further evaluation of responsiveness to change. We intend to investigate the differential changes in the disease acceptance scores within the individual time course of patients with different chronic diseases, and with respect to the differential use of distinct treatment strategies.

## Abbreviations

AKU questionnaire – AKU is an acronym of the German translation of "Adaptive Disease Coping"; ANOVA – analysis of variance; BDI – Beck-Depression-Index.

## Competing interests

Although the Oberberg Clinics value the therapeutic concept of an emotional disease acceptance, both the external authors of the University Witten/Herdecke, as well as the author from the Oberberg Clinic Schwarzwald were completely free to contribute without any political, personal, religious, ideological, academic, intellectual, commercial or any other interests. The authors did not receive financial support by organizations, companies etc. which could have influenced the interpretation of data. The preparation of the manuscripts was not financed by any organization.

## Authors' contributions

AB conceived the study, designed and developed the questionnaire, performed statistical analysis and drafted the manuscript. PFM contributed to develop the questionnaire and gave final approval of the manuscript. GM participated to conceive and design the study, to develop the questionnaire and contributed to draft the manuscript. All authors read and approved the final manuscript.
